# Campylobacteremia in stage IV gliosarcoma with bevacizumab treatment

**DOI:** 10.3402/jchimp.v2i1.17217

**Published:** 2012-04-30

**Authors:** Ping Liu

**Affiliations:** Sun Yat-Sen University of Medical Sciences, Guangzhou, Guangdong, China

**Keywords:** campylobacter bacteremia, immunocompromise, gliosarcoma, bevacizumab

## Abstract

Primary campylobacter enteritis with secondary bacteremia was diagnosed in an immunocompromised patient with stage IV gliosarcoma. She developed mild diarrhea followed by systemic symptoms with transient generalized weakness and fever. She was treated with azithromycin and had a full recovery and without relapse through 2 months of follow-up. Her diagnosis was confirmed by a positive stool culture for *Campylobacter sp*. and blood culture for *Campylobacter jejuni/coli*.

A 66-year-old Caucasian female with a stage IV gliosarcoma presented to an emergency department (ER) in Seattle, Washington in August 2011 because of generalized weakness.

The patient had been at her baseline state of health and able to ambulate independently until the evening of presentation when she was unable to arise from a chair. She denied headache and had no change in her baseline aphasia or right-sided weakness. She denied fever at home, chest pain, dyspnea, cough, abdominal pain, vomiting, and dysuria. She had recently had loose stools (once daily, no blood or mucous) for 5 days that resolved 1 day prior to admission. The patient had attributed her loose stools to laxative use for chronic constipation. Her past medical history included recurrent stage IV gliosarcoma, status post resection with subsequent brain radiation and chemotherapy with temozolomide in 2008 and repeat resection with brain radiation therapy in 2010. A recurrence in June 2011 was managed with bevacizumab, 10 mg/kg by intravenous infusion once every 2 weeks for 2 months with last dose given 10 days prior to admission, and concurrent dexamethasone starting at 16 mg/day and tapering to 4 mg by the evening prior to admission. Aside from the residual expressive aphasia and right-sided weakness related to her tumor recurrence, she also had impaired fasting glucose, chronic constipation, and dysphagia. Medications included omeprazole, levetiracetam, stool softeners, and senna. She previously worked as a nutritionist. She denied sick contacts, recent travel or exposure to new drugs. She denied consumption of undercooked meats, raw milk, herbs and other alternative substances but did have contact with raw poultry and other meats in the kitchen. She lived in a suburban neighborhood. Her only contact with animals was with a pet cat whose litter box she changed routinely.

Her temperature was 38.2°C, pulse rate 105/min, blood pressure 135/106 mmHg and respiration rate 20/min. She was well-nourished, developed, alert, and in no acute distress. Breath sounds were clear. Cardiac auscultation revealed mild tachycardia, regular rhythm, and no murmur. Abdomen was soft, non-tender and non-distended. The patient had baseline disorientation to place and time. Cranial nerves II through XII were grossly intact though she had moderate expressive aphasia without dysarthria or neglect. Muscle strength in extremities was symmetric bilaterally except for very slight right-sided pronator drift. DTRs were intact and symmetric. Finger-nose-finger testing was notable for slight dysmetria on the right upper extremity. The patient felt too weak to try to stand. There was no apparent sensory defect. The remainder of the examination was normal.

Her initial lab data (electrolytes, LFTs, lactate, urinalysis, CXR) were unremarkable aside from the CBC that showed mild leukopenia at WBC 3,700/µl, lymphopenia with lymphocytes 270/µl, granulocytes 3,182/µl and mild thrombocytopenia with platelets 142,000/µl. Two sets of blood cultures (3.5 hours apart) were collected.

On day 1 MRI of the brain without and with the administration of IV contrast showed no evidence of tumor progression and interval improved edema of the left frontal, parietal lobes and along the tumor resection margin. On day 2 the patient's generalized weakness resolved. She was able to ambulate independently. She remained afebrile. Blood cultures remained negative at 36 hours and so the patient was discharged. On day 3 morning the laboratory reported that one of the two sets of initial blood cultures on admission was growing small curved gram-negative rods (GNR), possibly campylobacter. The patient was then contacted and readmitted to the hospital for further evaluation. She had remained afebrile and denied recurrent weakness. No new symptoms developed. On readmission (day 3) she was more leukopenic (WBC 2,200/µl) and now neutropenic (granulocytes 920/µl) possibly related to the evolution of her untreated bacteremia, though her lymphopenia had resolved (lymphocytes 1,060/µl) and platelets were normal (167,000/µl). The patient started azithromycin 500 mg p.o. daily. It was thought that her transient generalized weakness and fever on day 1 may have been constitutional symptoms related to suspected campylobacter enteritis or mild manifestation of secondary bacteremia. Even though *Guillain-Barre syndrome* (GBS) may occur within days after the diarrheal episode, the patient had no new neurological findings on physical exam to suggest GBS. The patient remained asymptomatic and was discharged to home with suspected primary campylobacter enteritis with transient secondary bacteremia on a 10-day course of azithromycin 500 mg p.o. daily. She was educated for food safety and awareness of that her pet cat and the cat feces could be the source of campylobacter. Stool cultures collected on day 4 grew *Campylobacter* on day 6 and the GNR isolated from one set of day 1 blood cultures were also confirmed on day 13 to be *Campylobacter jejuni* versus *C. coli* (the testing method employed could not distinguish between these two organisms), sensitive to azithromycin. Repeat blood cultures from day 3 remained negative. After discharge the patient remained afebrile and had no weakness or diarrhea. A week later the CBC test normalized with WBC 5,100/µl (lymphocytes 1,160/µl, granulocytes 3,580/µl). Two months later her stool sample was negative for fecal lactoferrin and cultures negative for enteric pathogens. Refer to Table 1 for timeline of clinical events.

## Discussion

Campylobacter is the leading cause of human bacterial enteritis reported in the United States and other developed countries. Ingestion of contaminated undercooked poultry is the most common means of acquiring infection. Other common modes of transmission include ingestion of raw milk or untreated water, and contact with infected household pets (1). Campylobacter infection is characterized by diarrhea, fever, abdominal cramping and sometimes bloody stools. The constitutional symptoms often occur 12–24 hours before the onset of intestinal phase or may coincide with it in some patients or, less often, may follow it (2–4). In general, it causes mild, self-limiting enteritis in a normal host. Less frequent extraintestinal manifestations include bacteremia, focal infections such as septic arthritis (5), meningitis (6, 7), or endocarditis (8), and immune mediated processes such as reactive arthritis (9–11) and rarely *Guillain-Barre syndrome* (GBS) (12, 13).

The principal diarrheal pathogen is *C. jejuni*, which accounts for 80–90% of all cases of recognized illness due to *Campylobacter* species. The major species causing extraintestinal illnesses is *C. fetus*; however, any of the diarrheal agents may cause systemic or localized infection as well (14). Bacteremia has been estimated to occur in 0.1–1% of patients with *C. jejuni* or *coli* enteritis and almost always is transient and asymptomatic, but can be severe in immunosuppressed hosts (15). Most cases of bacteremia have been reported in patients with immune deficiency or other serious underlying conditions, including liver disease, hypogammaglobulinemia, malignancy, bone marrow and solid organ (liver and kidney) transplantation and human immunodeficiency virus (HIV) infection (1, 16–24), though bacteremia has also been described in previously healthy individuals (25). The patterns of bacteremia can be transient and self-resolving, or can be sustained, especially in immunocompromised patients with focal extra-intestinal infections (26). Data on mortality in cases of Campylobacteremia are limited, ranging from 4 to 16.4% for *C. jejuni* or *C. coli* (24, 25) and up to 28% for *C. fetus* (27). Antimicrobial therapy should be considered for *C. jejuni* infected patients who have bloody diarrhea, fever, worsening of symptoms, or a large number of stools, and in people who are immunosuppressed (28). Erythromycin or azithromycin are the drugs of choice (29, 30). The quinolones have been recommended as alternative therapy, but resistance to quinolones has been increasing in the last decade in most countries (31, 32). Interestingly, like our case, many cases of bacteremia have been shown to improve before receipt of appropriate antibiotics (25).

Our case is the first to report campylobacteremia associated with the treatment of the vascular endothelial growth factor inhibitor, bevacizumab. Although there were other risk factors for the development of campylobacteremia in our case, the potential role of bevacizumab should be considered because it could cause hypoxemia and necrosis of gastrointestinal (GI) epithelium, increasing the possibility of campylobacterial translocation from the GI tract into the blood stream. Other reported bevacizumab adverse reactions associated with GI damage include neutropenic infection at 54.8% and diarrhea at 21.4% in recurrent glioblastoma multiforme (33), sepsis from liver abscess infected with *Bacteroides fragilis* in colorectal carcinoma (34), GI ulceration (35) and perforation (36, 37).

Moreover, our case illustrates several important points about campylobacter enteritis and bacteremia. First of all, like our patient, most cases in the United States are domestically acquired *C. jejuni* infection. Potential exposures included her involvement in food preparation and caring for her pet cat. She denied consumption of raw milk, undercooked poultry or other meat. She may have been at higher risk due to her omeprazole, as proton-pump inhibitor use has been associated with a 4–13 fold increased risk of campylobacter enteric infection (38), and her immunocompromise and bevacizumab treatment likely increased her risk for bacteremia from enteric infection. However, it is surprising that our case was not more serious given all the risk factors the patient had. Secondly, our patient demonstrated that bacteremia can follow even mild enteritis, suggesting that blood cultures should be collected in patients at risk for complications. Thirdly, bacteremia can resolve and clinical improvement can occur prior to receipt of antibiotics, illustrating the importance of natural immunity, even in our immunocompromised patient. However, in some patients more serious complications such as sustained bacteremia and focal infections do occur, and thus all bacteremic patients with immunosuppression should be treated with systemic antibiotics (28). Finally, the slow growth of the organism in blood and stool cultures and the delayed final identification of the blood isolate in our patient are typical, consistent with the growth and fastidious nature of the campylobacter (1).

In summary, *Campylobacter* infection is common in the United States; however, bacteremia is an uncommon disease linked to immunosuppression and possibly in this case bevacizumab treatment. It typically presents with self-limiting enteric symptoms and acute febrile illness in a normal host but may cause a variety of extraintestinal manifestations under immunosuppression. Usually the prognosis is good with a full recovery on antimicrobial treatment. The internist must be aware of the possibility of bacteremia and complications from campylobacter infections, particularly in the immunocompromised population.

**Table 1:** Timeline

Diarrhea	5 days and resolved prior to admission
Generalized weakness	1 + day
Fever	Transient at ER
1st blood culture drawn on day 1	Negative
2nd blood culture drawn on day 1	*C. jejuni/coli* +
3rd blood culture drawn on day 3	Negative
4th blood culture drawn on day 3	Negative
Stool culture on day 4	Campylobacter +
Fecal lactoferrin on day 4	Positive
Azithromycin	500 mg p.o.×10 starting on day 3
Stool culture 2 months later	Negative
Fecal lactoferrin 2 months later	Negative
